# *Bacillus subtilis*-Fermented *Amomum xanthioides* Ameliorates Metabolic-Syndrome-Like Pathological Conditions in Long-Term HFHFD-Fed Mice

**DOI:** 10.3390/antiox11112254

**Published:** 2022-11-15

**Authors:** Jing-Hua Wang, Seung-Ju Hwang, Kwang-Soo Shin, Dong-Woo Lim, Chang-Gue Son

**Affiliations:** 1 Institute of Bioscience & Integrative Medicine, Daejeon University, 75, Daedeok-daero 176, Seo-gu, Daejeon 35235, Republic of Korea; 2 Liver and Immunology Research Center, Daejeon Korean Medicine Hospital, 75, Daedeok-daero 176, Seo-gu, Daejeon 35235, Republic of Korea; 3 Department of Microbiology, Graduate School, Daejeon University, Daejeon 34520, Republic of Korea; 4 Department of Diagnostics, College of Korean Medicine, Dongguk University, Dongguk-Ro 32, Goyang 10326, Republic of Korea

**Keywords:** *Bacillus subtilis*, *Amomum xanthioides*, fermentation, metabolic diseases, oxidative stress, gut microbiota

## Abstract

In modern society, numerous metabolic disorders are widespread globally. The present study aimed to demonstrate whether *Bacillus subtilis*-fermented *Amomum xanthioides* (BSAX) exerts anti-metabolic disturbance effects compared with the ethyl acetate fraction of *Amomum xanthioides* (EFAX), a previously verified functional fraction. Mice fed with a high-fat, high-fructose diet (HFHFD) for 10 wk presented a typical model of metabolic dysfunction, and BSAX significantly attenuated a string of metabolic-syndrome-related pathological parameters, such as body, fat, organ mass, lipid markers (TGs, TC, free fatty acids), and glucose metabolism (glucose, insulin), without influencing appetite. Further, BSAX markedly lowered malondialdehyde (MDA) and ROS in the blood and restored antioxidative parameters (SOD, GSH, and CAT in liver tissue, and total bilirubin in serum) by elevating Nrf2 and HO-1. Moreover, BSAX noticeably restored gut microbiota diversity and normalized lipid-metabolism-associated proteins, including SREBP-1, p-AMPK, and PPAR-α. Generally, most metabolic parameters were improved by BSAX to a greater extent than EFAX, except for liver weight and hepatic TC. In conclusion, BSAX alleviates metabolic dysfunction by enhancing lipid metabolism and antioxidative capacity and is more effective than EFAX. Therefore, the application of high-yield, effective BSAX might be a promising approach for curing and preventing metabolic disorders.

## 1. Introduction

Overconsumption of certain nutrients, as in a high-fat diet (HFD) or a high-fructose diet (HFrD), is one of the major causes of disruption to metabolic processes in the body [1]. Currently, the global prevalence rates of chronic metabolic diseases are high and rising fast [2], such as Type 2 diabetes (about 6.28% in 2017) [3], obesity (approximately 13% in 2016) [4], nonalcoholic fatty liver disease (NAFLD, nearly 30% in 2019) [5], and hypercholesterolemia (up to 39% in 2018) [6]. The epidemic of these chronic metabolic-related diseases has been imposing a heavy social and financial burden worldwide in recent decades [7,8]. Thus, looking for feasible therapeutic approaches and elucidating the associated mechanisms have become particularly imperative for responding to the global public health issue.

A large number of herbal medicines have been traditionally used for remedying metabolic disturbances, according to historical records in East Asia. As a widely known edible herbal medicine, *Amomum xanthioides* (AX, family Zingiberaceae) has previously been demonstrated to possess gastrointestinal-protective, hepatoprotective, anti-diabetic, anti-hyperlipidemia, and antibacterial effects [9]. Our previous study verified that the ethyl acetate fraction of *Amomum xanthioides* (EFAX) attenuated NAFLD by reducing lipid accumulation, excessive inflammation, and oxidative stress in an HFD-fed mouse model [10]. Fractionation by different solvent polarities is a common process for separating the ingredients in herbal extractions [11]. However, the widespread application of EFAX is restricted owing to its many limitations, such as the complicated fractionation processes, the low extraction rate (only 0.19%), and the toxic chemical residue. Accordingly, we have tried to find a feasible approach to overcome these issues.

On the other hand, fermentation has recently been deemed a proficient method for enhancing the original efficacy but reducing toxicity and has also been adopted to mitigate various metabolic disorders [11]. Numerous studies have shown that probiotic fermented herbs have various significantly heightened pharmacological activities relative to unfermented controls, e.g., for ameliorating obesity and endotoxemia [12,13,14]. *Bacillus subtilis* (*B. subtilis*) is a useful symbiotic microorganism and appears extensively in soil, plants, and the gastrointestinal tracts of humans, ruminants, and rodents [15]. Moreover, *B. subtilis* is famous for being dominant in a traditionally fermented soybean food called cheong-kuk-jang in Korea and natto in Japan. Recently, *B. subtilis* has been used in a wide range of applications in the food, medicine, and agricultural industries [16]. Several herbal medicines fermented using *B. subtilis* have various significantly enhanced pharmacological activities, such as reducing oxidative stress [17], anti-inflammation [18], and hyaluronic acid synthesis [19]. In addition, no significant cytotoxicity of AX extract was observed in several types of cell lines [20,21]. Our pilot in vitro test did not find B. subtilis-fermented AX (BSAX) to have cytotoxic effects on HepG2 and RAW264.7 cells, at least up to 100 μg/mL. Therefore, we postulated that BSAX might offer a solution for overcoming the limitations associated with multiple chemical fractionation procedures and also have pharmaceutical effects surpassing those of EFAX.

Hence, the present study aimed to compare the therapeutic effects of BSAX and EFAX against metabolic disorders using a long-term HFD and HFrD (HFHFD) consumption-induced mouse model. Meanwhile, related conceivable mechanisms, including oxidative stress suppression and gut microbiota regulation, were investigated to provide clues for developing healthy food and medicine.

## 2. Materials and Methods

### 2.1. Herbal Medicine Preparation

**Figure 1:**
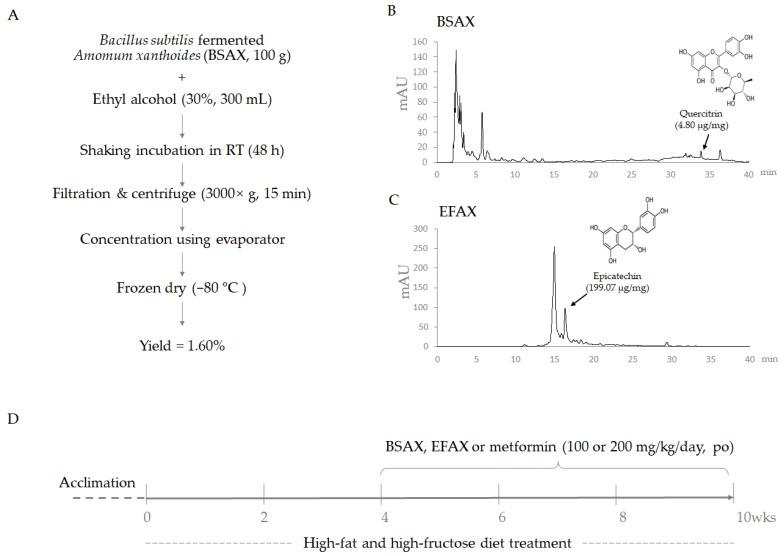
Procedure for obtaining the herbal extract, HPLC fingerprinting, and the experimental scheme. (**A**) Flowchart of the *Bacillus subtilis*-fermented *Amomum xanthioides* extraction (BSAX) process. (**B**,**C**) HPLC fingerprinting of BSAX and the ethyl acetate fraction of *Amomum xanthioides* (EFAX). (**D**) Animal experiment schedule.

Amomum xanthioides (Korean pharmacopeia standard) were obtained from the Jeong-Seong Pharmaceutical Company (Daejeon, Republic of Korea). Soil-origin Bacillus subtilis were cultured for 24 h in a shaking incubator at 37 °C and 150 rpm before fermentation. The Bacillus subtilis was confirmed by 16s ribosomal DNA sequencing (Table S1), using the Basic Local Alignment Search Tool (BLAST, https://blast.ncbi.nlm.nih.gov/Blast.cgi, accessed on 17 March 2022). Next, 500 g of Amomum xanthioides powder was mixed with 2.5 L MRS media (BD Difco, Marshfield, WI, USA). After autoclaving at 115 °C for 15 min, 2% of Bacillus subtilis (2 × 10^8^ CFU) was inoculated and incubated at 30 °C for one week without shaking. After that, 100 g of Bacillus subtilis-fermented Amomum xanthioides (BSAX) was added to 300 mL of 30% ethyl alcohol and shaken for 48 h. The residue was discarded after centrifugation at 3000× *g* for 15 min. The supernatant was dried with a lyophilizer (−80 °C), with a yield of 1.60% (*w*/*w*, Figure 1A). In addition, according to the procedure described in [10], the ethyl acetate fraction of Amomum xanthioides (EFAX) was fractionated, with a yield of 0.19% (*w*/*w*).

### 2.2. HPLC Fingerprinting Analysis

An Agilent 1260 high-performance liquid chromatograph (HPLC) with a bin pump, a degasser, an autosampler, a column oven, and a diode array detector (DAD) was applied for recording fingerprints. Both BSAX and EFAX were dissolved in 50% methanol. Epicatechin and quercitrin were used as inner reference compounds. The samples were eluted with a gradient of pure methanol and 2% acetic acid in distilled water in an Agilent Eclipse XDB-C18 column (4.6 mm × 250 mm, 5 μm) at a flow rate of 1 mL/min. Detection was carried out at 260 nm (Figure 1B,C).

### 2.3. Animal Experimental Design

Specific-pathogen-free C57BL/6j mice (6-week-old males) were commercially obtained from Daehan Biolink (Gyeonggi-do, Republic of Korea). During the whole experimental period, the mice had free access to the diet and were kept in conditions of 22 ± 2 °C and 50% ± 10% humidity. After one week of acclimatization, 30 mice were randomly divided into five groups (*n* = 6), and all the treatment groups were treated with a high-fat diet (HFD, D12492, 60% kcal% fat; Research Diet, New Brunswick, NJ, USA) and distilled-water (DW)-dissolved fructose (20% *w*/*v*, Sigma-Aldrich, Darmstadt, Germany), but the normal group was fed with a chow diet and DW. The present study used metformin as a positive control because it has a well-known capacity for improving the dysregulation of glucose metabolism. After four weeks of a high-fat and high-fructose diet (HFHFD), EFAX (200 mg/kg/day), BSAX (200 mg/kg/day), or metformin (100 mg/kg/day) were administered by oral gavage until the final experimental day (Figure 1D). The dosage of BSAX for animals was calculated based on the clinical dosage and body surface area. Meanwhile, DW was given to the normal and HFHFD groups via the same process as the drug-treated groups.

The consumption of food and water was noted once a week. The body weight was recorded in Weeks 5 and 10. The food efficiency ratio (FER) was calculated using the equation FER = (final body weight − initial body weight)/food intake × 100. Fecal samples were collected and stored at −40 °C the day before sacrifice. After 18 h of fasting, tail veins were punctured, and fasting blood glucose was determined with an ACCU-CHEK glucometer (Roche Diabetes Care GmbH, Mannheim, Germany). Blood from the abdominal aorta was collected under anesthesia on the final experimental day. An automatic hematology analyzer (Exigo Eos, Boule Medical AB, Spanga, Sweden) measured the complete blood count (CBC). The liver and fat tissues were removed and weighed, then the liver tissues were stored in 10% neutral formalin or −80 °C for further analysis. Three major types of adipose tissues, e.g., mesenteric fat, epididymal fat, and perirenal fat, were removed and weighed separately. Herein, a sum of the above fat weights was deemed as total white adipose tissue (WAT). The present study followed the Guide for Care and Use of Laboratory Animals (National Institutes of Health, 1996) and was approved by the Institutional Animal Care and Use Committee of Daejeon University (approval number: DJUARB-2022-013).

### 2.4. Biochemical Analysis in Serum and Hepatic Tissue

After 1 h of blood clotting, serum was separated by centrifugation at 3000× *g* for 15 min. Serum aspartate transaminase (AST), alanine transaminase (ALT), triglycerides (TGs), total bilirubin, and free fatty acid (FFAs) were determined with an Auto Chemistry Analyzer (Chiron, Emeryville, CA, USA). Serum insulin was measured using a mouse insulin ELISA kit, according to the manufacturer’s instructions (Invitrogen, Carlsbad, CA, USA). Hepatic TGs and total cholesterol (TC) were measured using commercial assay kits (ASAN Pharm, Anseong, Republic of Korea).

### 2.5. Hepatic Lipid Peroxidation Determination

Following a method described previously, 100 mg of liver tissue was homogenized with 1 mL of potassium chloride (11.5 mg/mL), and then 130 μL of liver homogenate was mixed with 80 μL of phosphoric acid and 260 μL of thiobarbituric acid (0.67%). After heating and centrifugation, the absorbance of the upper organic supernatant was determined at 535 and 520 nm. The final results were calculated using the 1,1,3,3-tetraethoxypropane (TEP) standard curve.

### 2.6. Determination of Serum and Hepatic Oxidative Stress and Antioxidative Parameters

A FastPrep-24 homogenizer (M.P. Biomedicals, Solon, OH, USA) was used to homogenize the frozen liver tissue with an RIPA buffer for the evaluation of hepatic reactive oxygen species (ROS), total antioxidant capacity (TAC), and other antioxidative parameters.

The serum and hepatic levels of ROS were determined using a method described previously [22]. Briefly, 5 μL of each liver tissue sample was mixed with 140 μL of a sodium acetate buffer (0.1 M) at room temperature. Next, 5 μL of N, N-diethyl-para-phenylenediamine (DEPPD, 10 mM) and 95 μL ferrous sulfate mixture (4.37 μM) was added for rapid reaction. After incubation of the mixture for 1 min, the ROS level was detected at 505 nm. Hydrogen peroxide (H_2_O_2_) was used to make a standard curve.

The TAC in serum or liver tissue was assessed using a protocol described previously [23]. Briefly, 90 μL of phosphate-buffered saline (PBS, 10 mM), 50 μL of myoglobin solution (18 μM), and 20 μL of 2,2′-azino-bis (3-ethylbenzthiazoline-6-sulfonic acid) diammonium salt (ABTS) solution (3 mM) were mixed with 10 μL serum or liver samples for 3 min of incubation. After 5 min of incubation with 20 μL of 30% H_2_O_2_, the absorbance was determined at 600 nm. A gallic acid solution was used as a standard.

The levels of hepatic superoxide dismutase (SOD), glutathione (GSH), and catalase (CAT) were determined using the SOD Assay Kit-WST (Dojindo Laboratories, Japan), the EZ-Glutathione Assay kit (DoGenBio, Seoul, Korea), and the EZ-Catalase Assay kit (DoGenBio, Seoul, Republic of Korea), respectively.

### 2.7. Histopathological Observation and Oil Red O Staining

The paraffin-embedded liver tissues were sliced to a thickness of 5 μm using a microtome (Leica RM225, Nussloch, Germany). After staining with hematoxylin and eosin, the sections were mounted in a rapid dry mounting medium (Biomeda, Foster City, CA, USA). Frozen liver tissues embedded in FSC22 clear frozen section medium (Leica, Germany) were sectioned in 5 μm thick slices and mounted on silicone-coated slides. All the prepared slides were stained with hematoxylin and eosin (H&E) or Oil Red O solution. After mounting and drying, an Olympus IX71 microscope (Tokyo, Japan) and DP70 camera (Tokyo, Japan) photographed the stained liver slides at 200× magnification. The area of red color after Oil Red O staining was quantified using ImageJ (National Institutes of Health, USA). NAFLD Activity Score (NAS) was evaluated by the sum of the steatosis grade (0–3), the lobular inflammation score (0–3), and the degree of hepatocyte ballooning (0–2).

### 2.8. Western Blotting

Six livers in each group were pooled together in the same proportions. The liver tissues homogenized in an RIPA (radio-immunoprecipitation assay) lysis buffer (Abcam, USA) were centrifuged for 5 min at 3000× *g*. The hepatic nuclear and cytosolic portions were separated using the Thermo Scientific NE-PER™ Nuclear and Cytoplasmic Extraction Reagents (cat. No.: 78,833; Rockford, IL, USA). The total protein concentration of the supernatant was measured with a BCA kit (Thermo Scientific, Waltham, MA, USA). Prepared liver samples (10 μL of 1 μg/μL for each lane) were separated in 10% polyacrylamide gel by electrophoresis and then transferred to a polyvinylidene fluoride (PVDF) membrane in the Mini-PROTEAN Tetra Cell System (BioRad, Hercules, CA, USA). After blocking, washing with Tween 20, and incubation with actin (MA 5-11869), GPAM (ab69990), SREBP-1 (ab28481), total AMPK-α (#5831), phospho-AMPK-α (#2535), PPAR-α (ab24509), Nrf2 (#12721), HO-1 (MA1-112), and Lamin B1 (ab16048) overnight at a 1/1000 dilution, the membranes were incubated with horseradish peroxidase-conjugated antibodies for 2 h (Table S2). The target bands were visualized using an advanced enhanced chemiluminescence reagent (Thermo Fisher, CA, USA) and photographed with a FUSION Solo Image system (Vilber Lourmat, Marne-la-Vallée, France). ImageJ (National Institutes of Health, Bethesda, MD, USA) was used to analyze the quantification of the bands.

### 2.9. Fecal Microbiota Analysis by 16s rRNA Gene Amplicon Sequencing

Briefly, fecal metagenomic DNA was extracted using a QIAamp DNA stool Mini Kit (Qiagen, Hilden, Germany), according to the manufacturer’s instructions. After quality checking by 1% agarose gel electrophoresis, the 16s rRNA gene (V3–V4 region) primers targeting 341F and 805R were used for bacterial PCR amplification. The amplified bacterial DNA was sequenced by a commercial service from Chunlab, Inc. (Seoul, Republic of Korea) using a Miseq sequencing system (Illumina, San Diego, CA, USA). After deletion of the low-quality reads, which had average quality scores of <25 and read lengths of <300 bps, the trimmed sequences were denoised using DUD-Seq software. The quality-controlled sequences were used for taxonomic assignment. Furthermore, CL community software was used to analyze alpha and beta diversity (Chunlab, Seoul, Republic of Korea).

### 2.10. Statistical Analysis

Data were analyzed statistically using the Statistical Package for the Social Sciences (SPSS, version 19.0, Chicago, IL, USA) software, and the data are presented as the means ± SDs (standard deviations). A one-way ANOVA was performed following the least significant difference (LSD) post hoc test to determine the significant differences. *p*-values less than 0.05 were considered statistically significant.

## 3. Results

### 3.1. BSAX Exerted Anti-Obesity and Anti-Hyperlipidemic Effects Superior to Those of EFAX

**Figure 2:**
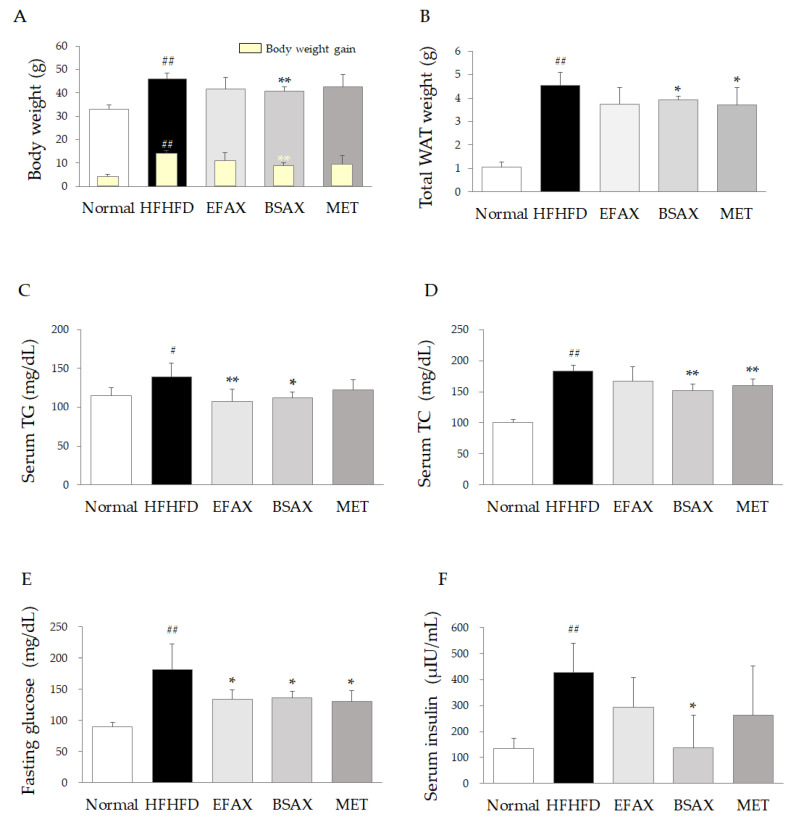
BSAX mitigated HFHFD-induced metabolic disturbance. (**A**,**B**) Final body weight (the body weight gain during the drug treatment is shown in yellow) and total white adipose tissue (WAT) weight were recorded on the last experimental day. (**C**,**D**) Serum triglycerides (TGs) and total cholesterol (TC) were determined using a Chemistry Auto Analyzer (Chiron, Emeryville, CA, USA). (**E**) After 18 h of fasting, fasting blood glucose (FBG) was detected using a drop of blood from the tail end. (**F**) Serum insulin levels were measured with the mouse insulin ELISA kit (Invitrogen, Carlsbad, CA, USA). ^#^ *p* < 0.05, ^##^ *p* < 0.01, compared with the normal group; * *p* < 0.05, ** *p* < 0.01, compared with the HFHFD control.

After the 10-week HFHFD treatment, the HFD and fructose intakes were markedly reduced compared with the chow diet (*p* < 0.01 or 0.05, Table 1), and no difference was observed among the four HFHFD groups, including the BSAX and metformin control group. After 10 weeks of the HFHFD, body (gain) and WAT weights were dramatically increased compared with the normal group (*p* < 0.01), while they were significantly attenuated by the 6-week BSAX treatment compared with the control (*p* < 0.01 or 0.05, Figure 2A,B). BSAX significantly reduced the FER compared with EFAX and metformin (*p* < 0.01, Table 1). Metformin reduced WAT weight but not body weight (gain) compared with the HFHFD group (*p* < 0.01, Figure 2A,B).

### 3.2. BSAX Attenuated Abnormalities in Hepatic Enzymes and Lipid Parameters More Than EFAX

As expected, the 10-week HFHFD treatment perceptibly elevated serum levels of AST, ALT, TG, TC, and FFA compared with the chow diet (*p* < 0.05 or 0.01). BSAX treatment markedly attenuated these alterations, while EFAX significantly reduced only the alteration in serum TG levels (*p* < 0.05 or 0.01, Table 1). Although metformin treatment showed a slight tendency to improve these alterations, the improvements did not reach statistical significance.

### 3.3. BSAX Attenuated Fasting Blood Hyperglycemia and Hyperinsulinemia

The 10-week HFHFD notably elevated fasting blood glucose, fasting serum insulin, and HOMA-IR scores compared with the normal group (*p* < 0.05); however, BSAX significantly attenuated these elevations compared with the HFHFD control (*p* < 0.05 or 0.01, Figure 2E,F and Table 1). In particular, BSAX was more effective than EFAX and metformin in inhibiting fasting serum insulin and HOMA-IR.

### 3.4. BSAX Restored Platelets, Leukocytes, and Lymphocytes

The 10-week HFHFD treatment significantly increased platelet numbers and noticeably decreased total numbers of leukocytes (mainly in lymphocytes and granulocytes) compared with the normal group (*p* < 0.01, Table 2). These alterations in platelet and leukocyte counts were notably restored by BSAX (*p* < 0.01, Table 2). Metformin also showed a similar effect on platelets and leukocytes to BSAX, but EFAX only significantly restored the leukocyte count.

### 3.5. BSAX Attenuated Hepatic Steatosis More Than EFAX

**Figure 3:**
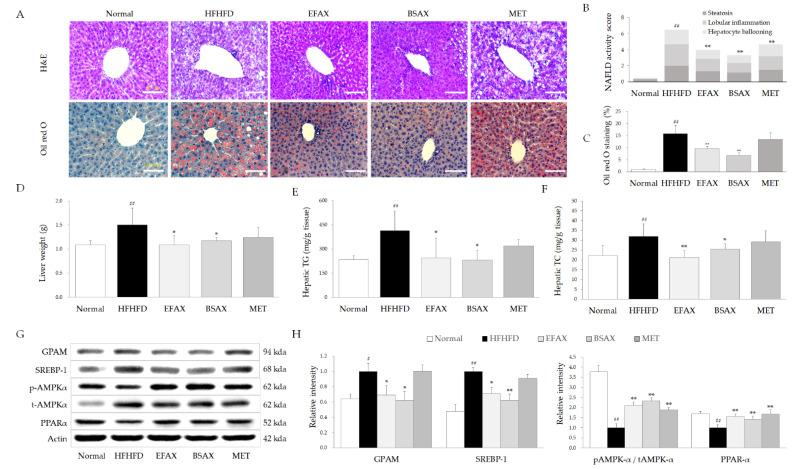
BSAX ameliorated hepatic steatosis by inhibiting lipogenesis. (**A**) Hematoxylin and eosin (H&E) staining and Oil Red O (ORO) staining were carried out on the liver tissues. (**B**) Nonalcoholic fatty liver disease activity scores (NASs) were calculated for comparison. (**C**)The area of red in the ORO staining was analyzed using ImageJ v1.8.0 (NIH, Bethesda, MD, USA). (**D**) The livers were removed and weighed immediately at the end of the experimental period. (**E**,**F**) The hepatic triglyceride (TG) and total cholesterol (TC) levels were measured with a Chemistry Auto Analyzer (Chiron, Emeryville, CA, USA). (**G**) The levels of hepatic lipid-metabolism-associated proteins were assessed by Western blotting. (**H**) ImageJ v1.8.0 was used to quantify the relative intensities of the bands. ^#^ *p* < 0.05, ^##^ *p* < 0.01, compared with the normal group; * *p* < 0.05, ** *p* < 0.01, compared with the HFHFD control.

The 10-week HFHFD treatment increased liver weights by approximately 1.3-fold compared with the normal group (*p* < 0.01), which were significantly normalized by both BSAX and EFAX (*p* < 0.01 or 0.05, Figure 3D). As expected, both the BSAX and EFAX treatments also perceptibly attenuated the wide areas of fat vacuoles and hepatocyte swelling, as well as excessive lipid droplets in hepato-histopathological observations compared with the HFHFD group, along with NAFLD activity score (NAS) (*p* < 0.05 or 0.01, Figure 3A–C).

In addition, the HFHFD-induced increases in hepatic TG and TC levels were significantly attenuated by BSAX and EFAX (*p* < 0.05 or 0.01, Figure 3E,F). Moreover, both BSAX and EFAX significantly normalized the altered protein activity levels of hepatic glycerol-3-phosphate acyltransferase (GPAM), sterol regulatory element-binding protein 1 (SREBP-1), and hepatic peroxisome proliferator-activated receptor alpha (PPARα) and the phosphorylation of AMPKα (Figure 3G,H). Metformin also showed a tendency to improve all parameters but was inferior to BSAX or EFAX.

### 3.6. BSAX Attenuated Oxidative Stress More Than EFAX

**Figure 4:**
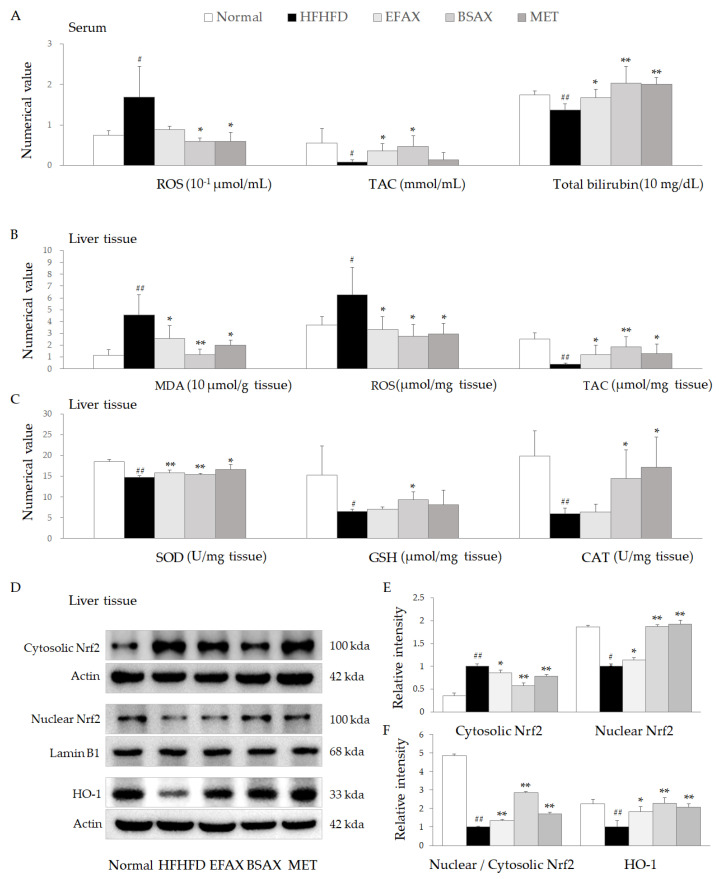
BSAX attenuated systemic and hepatic oxidative stress through the restoration of antioxidant capacity. (**A**) The levels of ROS, TAC, and total bilirubin in sera were determined according to the appropriate methods. (**B**,**C**) The levels of hepatic MDA, ROS, TAC, and the main antioxidative parameters (SOD, GSH, CAT) were also evaluated for comparison. (**D**) The levels of oxidative stress regulator-related proteins in hepatic tissue were detected by Western blotting. (**E**,**F**) ImageJ v1.8.0 was used for quantification of the relative intensities of the bands. ^#^ *p* < 0.05, ^##^ *p* < 0.01, compared with the normal group; * *p* < 0.05, ** *p* < 0.01, compared with the HFHFD control. ROS, reactive oxygen species; TAC, total antioxidant capacity; MDA, malondialdehyde; SOD, superoxide dismutase; GSH, glutathione; CAT, catalase.

The 10-week HFHFD treatment significantly increased ROS in peripheral serum and suppressed systemic TAC and total bilirubin levels, while these were significantly attenuated by BSAX, and EFAX showed slightly inferior activity (*p* < 0.05 or 0.01, Figure 4A).

As expected, the HFHFD noticeably elevated MDA and ROS in liver tissues and had a tendency to lower antioxidative component activities, including TAC, SOD, GSH, and CAT. Meanwhile, BSAX (and EFAX, partially) significantly attenuated the oxidative stressor makers and restored the depletion in antioxidant components (*p* < 0.05 or 0.01, Figure 4B,C). These antioxidant effects of BSAX and EFAX were supported by the results for protein levels of nuclear factor erythroid 2-related factor 2 (Nrf2) in hepatic nuclear portions and heme oxygenase-1 (HO-1) in the liver tissues (*p* < 0.05 or 0.01, Figure 4D–F). The overall effects of metformin were similar to those of BSAX and EFAX.

### 3.7. BSAX Expanded the Diversity of Gut Microbiota

**Figure 5:**
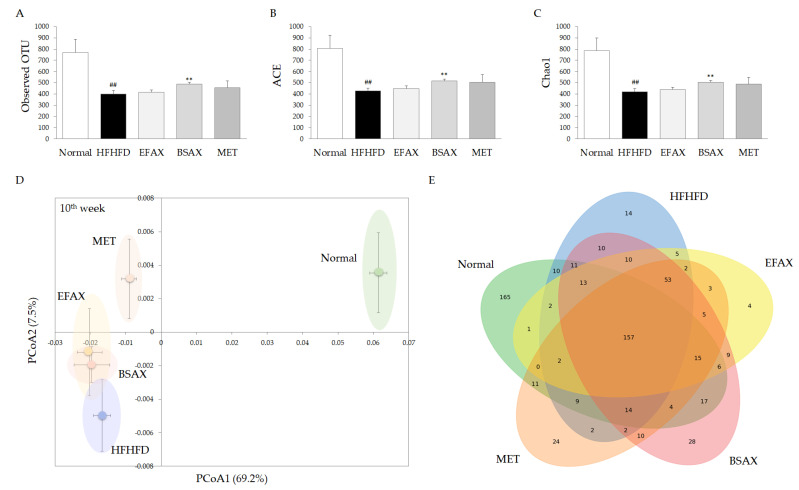
The gut microbiota was significantly resilient and was recovered by BSAX. (**A**–**C**) Alpha diversity indices (observed OTU, ACE, and Chao1 indices) for the gut microbiota analysis. (**D**) PcoA analysis of the fecal 16s rDNA sequencing data was conducted and diagramed to evaluate the similarities among the groups. (**E**) The Venn diagram illustrates the number of each group’s shared and unique species in the gut microbiota. ^##^ *p* < 0.01, compared with the normal group; ** *p* < 0.01, compared with the HFHFD control.

The analysis of alpha diversity in the fecal microbiota showed that the 10-week HFHFD treatment perceptibly reduced the observed OTUs by approximately 43.6% in the normal group and reduced species richness levels (ACE and Chao1 indices). However, BSAX (but not EFAX) significantly restored the observed OTU, ACE, and Chao1 indices compared with the HFHFD control (*p* < 0.01, Figure 4A–C). The beta diversity results showed obvious differences in the microbial communities between the normal and other groups (Figure 5D). Unlike metformin, BSAX and EFAX slightly changed the microbial communities compared with the HFHFD control (Figure 5D). Alongside 157 commonly shared core microbial species in all groups, 165 unique microbial species were found in the normal group. Meanwhile, only 14, 28, 4, and 24 microbial species were found uniquely in the HFHFD, BSAX, EFAX, and metformin groups, respectively (Figure 5E).

## 4. Discussion

In modern society, metabolic disturbance, commonly referred to as comorbidity with NAFLD, hyperlipidemia, obesity, and Type 2 diabetes, has become increasingly serious worldwide [1,24]. It has already been proven that a long-term HFHFD treatment is a reliable approach for establishing an animal model of chronic metabolic disorders [25,26]. Unlike the previous HFD-only model, animal models fed with a combination of an HFD and a high-fructose diet have been more frequently applied due to the very humanlike clinical symptoms exhibited [27]. As we expected, 10 wk of free HFHFD intake markedly elevated body and fat weight, hypertriglyceridemia, hypercholesterolemia, hyperglycemia, and hyperinsulinemia in the present study.

The liver is a central organ that keeps the metabolic balance between a lack and an overload of nutrients, including lipids and glucose [28]. As shown in the present results, chronic overnutrition induces metabolic disturbances in multiple tissues, including fat, pancreas, and liver tissues [29]. In overweight and obese subjects, liver fat is positively correlated with AST and ALT serum levels, indicating liver injury (r > 0.7, *p* < 0.001) [30]. In line with our previous report [10], EFAX also significantly attenuated HFHFD-induced hepatic steatosis and inflammation and had actions on serum TG and fasting blood glucose levels (*p* < 0.05 or *p* < 0.01, Figure 2). On the other hand, BSAX showed more effective actions on the alterations described above than EFAX, and only BSAX (but not EFAX) significantly ameliorated the increases in body and fat weight (*p* < 0.05 or *p* < 0.01, Figure 2). Moreover, EFAX has a very low yield (0.19%) after a multi-step extraction procedure, while the fermentation process is relatively simple, yielding a relatively high amount of product (1.60%) at a lower cost and using no toxic solvent to ensure safety. Accordingly, BSAX might be a promising route for the development of pharmaceutical resources using AX to prevent and improve metabolic dysfunction.

The accumulated data support the pathological contributions of nutritional overload and consequent obesity to further systemic risk factors, such as chronic kidney disease, cardiovascular disease, and metabolic-syndrome-like disorders [31,32]. These harmful obesity-related influences result from a recent maladaptation of the human body to rapid overnutrition in contrast to the background of long-sustained contrary circumstances [32]. The evolution of the human genome was adapted to a lack of food/nutrition for millions of years, and our body has difficulty dealing with the overnutrition state [33]. Many chronic diseases are related to overnutrition, and more than 75% of all deaths are attributed to an unbalanced diet and overnutrition in developed countries [34]. This trend is rapidly increasing in developing countries as well [34]. Accordingly, the development of therapeutics to combat metabolic disorders is urgently required.

As a typical overnutrition-derived disease, the prevalence of NAFLD was nearly 30% worldwide in 2019 [5]. Nevertheless, no specific drug for NAFLD has been approved by the FDA yet [35]. In our study, HFHFD completely induced hepatic steatosis, as shown by the pathophysiological observations and lipid staining of the liver tissues. Meanwhile, the increased NAFLD activity scores, serum AST and ALT levels, and hepatic TG and TC levels also demonstrated that hepatocyte and lipid metabolism was impaired in the liver. Whereas both EFAX and BSAX noticeably inhibited abnormal lipid accumulation in the liver tissues, ameliorating liver function, BSAX slightly surpassed EFAX in ameliorating hepatic lipid accumulation without a significant difference.

These BSAX-derived effects on NAFLD were also repeated for glucose metabolism, as measured by fasting glucose, serum insulin, and HOMA-IR (Figure 2 and Table 1). The pharmaceutical actions of BSAX were supported by the changes in the key molecules involved in lipid and insulin signaling, including SREBP-1, GPAM, and AMPK (Figure 3). SREBP-1 is an essential transcriptional regulator for lipogenesis after the response to insulin signaling [36]. The HFHFD-induced excessive blood glucose promoted insulin secretion and eventually led to insulin resistance, which triggered lipogenesis and lipid accumulation through SREBP-1 and GPAM [37]. In addition, phosphorylation of AMPK, a pivotal regulator of energy metabolism, can suppress the processes of metabolic disorders, such as NAFLD, obesity, and Type 2 diabetes [38]. The clinical meta-analysis revealed that metformin significantly reduced body mass, TC, and TGs in Type 2 diabetic subjects [39]. Therefore, as a first-line antidiabetic and AMPK activator, metformin was used as a positive control in the present study. However, metformin was only effective in phosphorylating AMPK rather than regulating SREBP-1 and GPAM. Thus, the present results might indicate that BSAX and EFAX ameliorate glucose and lipid metabolism together, such that they are superior to metformin.

Meanwhile, oxidative stress is due to an imbalance in oxidative stressors and antioxidative components [40]. Excessive oxidative stress is crucial in causing metabolic dysfunction, including insulin resistance, dyslipidemia, etc. [41,42]. Clinical survey data have also shown lower antioxidants in adults with metabolic syndrome compared with normal subjects [43]. BSAX markedly attenuated systemic and hepatic oxidative stress by restoring antioxidative factors, such as total bilirubin, SOD, GSH, and CAT (Figure 4). In addition, transcription factor nuclear factor erythroid 2-related factor 2 (Nrf2) positively enhanced antioxidant defenses and ameliorated various metabolic diseases [44]. The conjugated form of Nrf2 directly binds to the antioxidant response element (ARE) in the nucleus and increases gene transcription of HO-1 [45]. Subsequently, HO-1 accelerates heme degradation to produce more bilirubin, exerting an antioxidative effect [45]. Therefore, BSAX activation of the Nrf2/HO-1 signaling pathway can be estimated as a potential mechanism for relieving metabolic disorders by regulating oxidative stress.

On the other hand, BSAX was more effective at normalizing platelets and leukocytes than EFAX and metformin. Previous clinical observations [46,47] and the present data have collectively shown that HFHFD significantly increases platelet numbers and platelet–lymphocyte ratios (PLR, Table 2). A distinct increase in PLR was also observed in subjects with later-stage diabetes [48]. Therefore, improvements in leukopenia (mainly caused by lymphopenia and granulopenia) and thrombocythemia by BSAX might be potential causes of the improved metabolic disturbances. Meanwhile, more and more studies have verified that the commensal gut microbiota is essential in modulating the host’s metabolism [49]. So far, human genetic manipulation is difficult and extremely dangerous in the clinic. Therefore, more and more scholars believe that regulation of gut microbiota could be a promising approach for ameliorating human metabolic disturbances [50]. We found that only BSAX markedly recovered the numbers of gut bacterial species as compared with EFAX and metformin (Figure 5A–C). However, we did not find an obvious difference in overall gut microbiota structures between the BSAX and EFAX groups (Figure 5D). A European cohort study revealed that insulin resistance and blood glucose levels are associated with lower microbial diversity in the human gut [51]. In addition, gut microbiota diversity was found to be low in obese subjects [52]. Thus, enhancement of gut microbiota diversity by BSAX is a prospective way to regulate the host’s metabolism, especially for glucose and lipids. The statistical results indicated that particular phyla and genera are strongly correlated with oxidative stress markers and antioxidative parameters (Figure S2). These will provide valuable clues for studying the effects of gut microbiota and oxidative stress under overnutrition-induced metabolic-syndrome-like pathological conditions in the future.

From the present data, we found that EFAX had anti-fatty liver effects, similar to previous reports for NAFLD [10] and hepatic fibrosis [53]. Meanwhile, BSAX not only beneficially regulated hepatic lipid metabolism but also effectively modulated metabolic dysfunction with respect to systematic lipids and glucose (Figure 2). Thus, the BSAX is appropriate for comprehensive metabolic disorders, such as metabolic syndrome. The HPLC profile indicated predominantly different chemical components between BSAX and EFAX (Figure 1B,C). We also noted that epicatechin was not detected, and that quercitrin was relatively high in BSAX compared with EFAX. Therefore, one of the possible explanations is the change in chemical constituents after microbial bioconversion. However, the current study has limitations in terms of identifying the complete components. An exploration of the effective components and optimization of the fermentation conditions will be undertaken in future studies.

## 5. Conclusions

We can conclude that BSAX is more effective in alleviating metabolic-syndrome-like pathological conditions by ameliorating lipid metabolism, enhancing antioxidative capacity, and restoring gut microbiota. Soil-isolated *B. subtilis* is a reliable probiotic for enhancing the pharmaceutical effects of herbal medicine through bioconversion. Our results support the idea that *B. subtilis*-fermented AX might be more applicable than fractionated AX as a promising candidate for remedying and preventing multiple metabolic disorders.

## Figures and Tables

**Table 1:** Food and fructose consumption and biochemical parameters of sera.

Contents	Normal	HFHFD	EFAX	BSAX	MET
Food intake (g/day/mouse)	3.21 ± 0.07	1.99 ± 0.11 ^##^	1.94 ± 0.08	1.90 ± 0.06	1.88 ± 0.10
Fructose intake (mL/week/mouse)	12.8 ± 1.0	11.3 ± 1.2 ^#^	11.0 ± 1.0	11.0 ± 0.3	10.5 ± 0.8
FER (%)	1.15 ± 0.20	6.33 ± 0.82 ^##^	5.10 ± 1.43	4.12 ± 0.70 **	4.56 ± 1.84
AST (IU/L)	50.8 ± 5.5	83.3 ± 13.7 ^##^	71.0 ± 13.8	61.4 ± 6.8 *	66.6 ± 9.5 *
ALT (IU/L)	16.4 ± 2.0	48.2 ± 17.2 ^##^	31.3 ± 14.7	29.9 ± 5.3 *	31.5 ± 13.8
FFA (mEq/L)	0.88 ± 0.11	1.02 ± 0.01 ^#^	0.89 ± 0.09 *	0.91 ± 0.04 **	1.01 ± 0.22
HOMA-IR	0.54 ± 0.19	3.44 ± 1.11 ^##^	1.78 ± 0.88 *	0.85 ± 0.83 **	1.52 ± 1.24 *

^#^ *p* < 0.05, ^##^ *p* < 0.01, compared with the normal group; * *p* < 0.05, ** *p* < 0.01 compared with the HFHFD group; n = 6. HFHFD, high-fat and high-fructose diet; EFAX, ethyl acetate fraction of *Amomum xanthoides*; BSAX, *Bacillus subtilis*-fermented *Amomum xanthoides*; MET, metformin; FER, food efficiency ratio; AST, aspartate aminotransferase; ALT, alanine aminotransferase; FFA, free fatty acid; HOMA-IR, homeostatic model assessment for insulin resistance.

**Table 2:** Hematological parameters.

Hematological Indices	Normal	HFHFD	EFAX	BSAX	MET
Erythrocytes (10^9^/mL)	8.0 ± 0.7	8.2 ± 0.4	8.4 ± 0.6	8.7 ± 0.8	8.8 ± 0.7
Hemoglobin (g/dL)	12.1 ± 1.1	12.5 ± 0.5	13.0 ± 1.1	13.3 ± 1.1	13.7 ± 1.2
Platelets (10^6^/mL)	264 ± 147	630 ± 227 ^##^	409 ± 314	377 ± 104 *	300 ± 146 *
Leukocytes (10^6^/mL)	13.4 ± 3.5	6.1 ± 1.2 ^##^	9.1 ± 2.8 *	14.0 ± 2.6 **	12.3 ± 3.7 **
Lymphocytes (10^6^/mL)	9.2 ± 2.3	4.3 ± 1.8 ^##^	6.9 ± 2.0 *	10.9 ± 1.9 **	9.4 ± 3.1 *
Platelet–lymphocyte ratio	31.0 ± 17.7	163.2 ± 68.2 ^##^	60.3 ± 36.4 *	34.8 ± 9.8 **	34.6 ± 21.4 **
Monocytes (10^6^/mL)	0.9 ± 0.4	0.5 ± 0.1	0.6 ± 0.2	0.9 ± 0.2 **	0.8 ± 0.2 **
Granulocytes (10^6^/mL)	3.4 ± 1.0	1.8 ± 0.2 ^#^	1.7 ± 0.7	2.3 ± 0.5 *	2.1 ± 0.4

^#^ *p* < 0.05, ^##^ *p* < 0.01, compared with the normal group; * *p* < 0.05, ** *p* < 0.01, compared with the HFHFD group; n = 6. HFHFD, high-fat and high-fructose diet; EFAX, ethyl acetate fraction of *Amomum xanthoides*; BSAX, *Bacillus subtilis*-fermented *Amomum xanthoides*; MET, metformin.

## Data Availability

The data is contained within the article and the Supplementary Materials.

## Supplementary Materials

The following are available online at https://www.mdpi.com/article/10.3390/antiox11112254/s1. Table S1: Microbial identification by 16s ribosomal RNA sequencing; Table S2: Western blot antibody information; Figure S1: Data of the rarefaction curve; Figure S2: Correlation between gut microbiota and oxidative-stress-related parameters.
