# Supplementary material for: Bacillus subtilis-Fermented Amomum xanthioides Ameliorates Metabolic-Syndrome-Like Pathological Conditions in Long-Term HFHFD-Fed Mice
Source: Antioxidants (Basel). 2022 Nov 15;11(11):2254. doi: 10.3390/antiox11112254 (PMC9687221; doi:10.3390/antiox11112254)
Supplement: Supplementary file 1 [file antioxidants-11-02254-s001.zip › antioxidants-1942617-supplementary.pdf]

Table S1. Microbial identification by 16s ribosomal RNA sequencing

Sample name: BS211229

Number of bases: 1492 bps GC: 50.8% AT: 49.2%

Primer: 27F and 1429R

GCTTGCTCCCTGATGTTAGCGGCGGACGGGTGAGTAACACGTGGGTAACCTGCCTGTAAGACTGGGATAACTCCGGGAAACCGGGGCTAATACCGGATGGTTGTTTGAACCGCATGGT
TCAGACATAAAAGGTGGCTTCGGCTACCACTTACAGATGGACCCGCGGCGCATTAGCTAGTTGGTGAGGTAACGGCTCACCAAGGCGACGATGCGTAGCCGACCTGAGAGGGTGATC
GGCCACACTGGGACTGAGACACGGCCCAGACTCCTACGGGAGGCAGCAGTAGGGAATCTTCCGCAATGGACGAAAGTCTGACGGAGCAACGCCGCGTGAGTGATGAAGGTTTTCG
GATCGTAAAGCTCTGTTGTTAGGGAAGAACAAGTGCCGTTCAAATAGGGCGGCACCTTGACGGTACCTAACCAGAAAGCCACGGCTAACTACGTGCCAGCAGCCGCGGTAATACGTA
GGTGGCAAGCGTTGTCCGGAATTATTGGGCGTAAAGGGCTCGCAGGCGGTTTCTTAAGTCTGATGTGAAAGCCCCCGGCTCAACCGGGGAGGGTCATTGGAAACTGGGGAACTTGAG
TGCAGAAGAGGAGAGTGGAATTCCACGTGTAGCGGTGAAATGCGTAGAGATGTGGAGGAACACCAGTGGCGAAGGCGACTCTCTGGTCTGTAACTGACGCTGAGGAGCGAAAGCG
TGGGGAGCGAACAGGATTAGATACCCTGGTAGTCCACGCCGTAAACGATGAGTGCTAAGTGTTAGGGGGTTTCCGCCCCTTAGTGCTGCAGCTAACGCATTAAGCACTCCGCCTGGGG
AGTACGGTCGCAAGACTGAAACTCAAAGGAATTGACGGGGGCCCGCACAAGCGGTGGAGCATGTGGTTTAATTCGAAGCAACGCGAAGAACCTTACCAGGTCTTGACATCCTCTGA
CAATCCTAGAGATAGGACGTCCCCTTCGGGGGCAGAGTGACAGGTGGTGCATGGTTGTCGTCAGCTCGTGTCGTGAGATGTTGGGTTAAGTCCCGCAACGAGCGCAACCCTTGATCTT
AGTTGCCAGCATTCAGTTGGGCACTCTAAGGTGACTGCCGGTGACAAACCGGAGGAAGGTGGGGATGACGTCAAATCATCATGCCCCTTATGACCTGGGCTACACACGTGCTACAAT
GGACAGAACAAAGGGCAGCGAAACCGCGAGGTTAAGCCAATCCCACAAATCTGTTCTCAGTTCGGATCGCAGTCTGCAACTCGACTGCGTGAAGCTGGAATCGCTAGTAATCGCGG
ATCAGCATGCCGCGGTGAATACGTTCCCGGGCCTTGTACACACCGCCCGTCACACCACGAGAGTTT

BLASTN 2.2.30+ Database: Nucleotide collection (nt) 57,030,965 sequences; 257,768,224,489 total letters

Reference: Zheng Zhang, Scott Schwartz, Lukas Wagner, and Webb Miller (2000), "A greedy algorithm for aligning DNA sequences", J Comput Biol 2000; 7(1-2):203-14.

Description	Scientific Name	Max Score	Total Score	Query Cover	Percent Identity	Accession Length	Accession
Bacillus subtilis strain JCM 1465 16S ribosomal RNA, partial sequence	Bacillus subtilis	2466	2466	100%	99.63	1472	NR_113265.1
Bacillus subtilis strain NBRC 13719 16S ribosomal RNA, partial sequence	Bacillus subtilis	2466	2466	100%	99.63	1475	NR_112629.1
Bacillus subtilis strain DSM 10 16S ribosomal RNA, partial sequence	Bacillus subtilis	2466	2466	100%	99.63	1517	NR_027552.1
Bacillus subtilis strain IAM 12118 16S ribosomal RNA, complete sequence	Bacillus subtilis	2460	2460	100%	99.56	1550	NR_112116.2
Bacillus subtilis strain BCRC 10255 16S ribosomal RNA, partial sequence	Bacillus subtilis	2460	2460	100%	99.56	1468	NR_116017.1
Bacillus subtilis strain NCDO 1769 16S ribosomal RNA, partial sequence	Bacillus subtilis	2416	2416	100%	98.96	1427	NR_118972.1
Bacillus subtilis strain SBMP4 16S ribosomal RNA, partial sequence	Bacillus subtilis	2399	2399	100%	98.74	1463	NR_118383.1
Bacillus subtilis strain NRRL NRS-744 16S ribosomal RNA, partial sequence	Bacillus subtilis	2130	2130	86%	99.57	1168	NR_116192.1
Bacillus subtilis strain NRRL B-4219 16S ribosomal RNA, partial sequence	Bacillus subtilis	2130	2130	86%	99.57	1168	NR_116183.1

Company: Cosmogenetech co, Ltd.

Website: www.cosmogenetech.com

Table S2. Western blot antibodies information

Peptide/Protein target	Manufacturer	Catalog	Dilution ratio
Actin	Thermo Fisher scientific	MA 5-11869	1:1,000
GPAM	abcam	ab69990	1:1,000
SREBP-1	abcam	ab28481	1:1,000
Total AMPK-α	Cell signaling technology	#5831	1:1,000
Phospho AMPK-α	Cell signaling technology	#2535	1:1,000
PPAR-α	abcam	ab24509	1:1,000
Nrf2	Cell signaling technology	#12721	1:1,000
Lamin B1	abcam	ab16048	1:1,000
HO-1	Thermo Fisher scientific	MA1-112	1:1,000
Goat Anti-Rabbit IgG (HRP)	GeneTex	GTX213110-01	1:5,000
Goat Anti-Mouse IgG (HRP)	GeneTex	GTX213111-01	1:5,000

Abbreviations: GAMP, Glycerol-3-Phosphate Acyltransferase, Mitochondrial; PPAR- α, Peroxisome proliferator-activated receptor; AMPK, AMP-activated protein kinase; FAS, fatty acid synthase; SREBP-1, Sterol regulatory element-binding protein 1; Nrf2, Nuclear factor E2-related factor; HO-1, Heme oxygenase-1; HRP, horseradish peroxidase; IgG, Immunoglobulin G.

Figure S1. Data of rarefaction curve

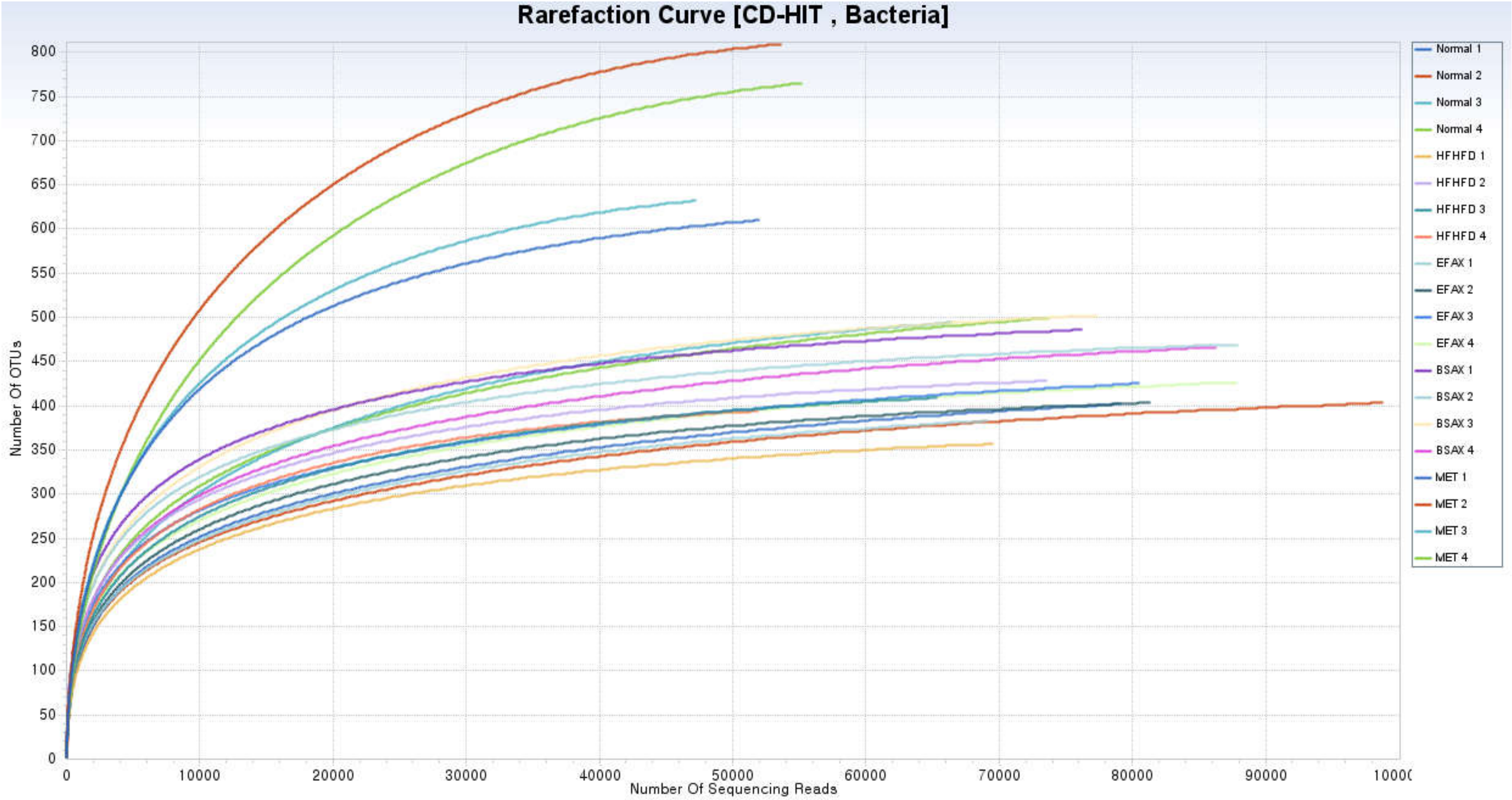

Figure S2. Correlation between gut microbiota and oxidative stress-related parameters

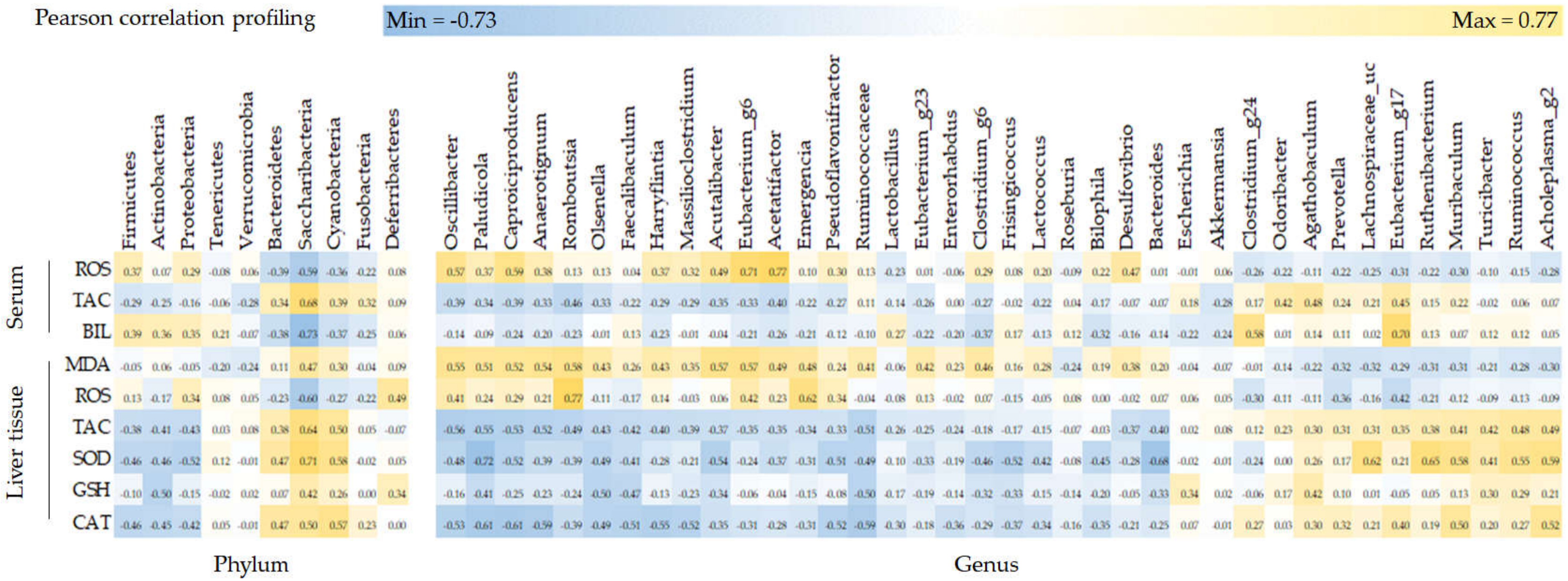

The two-tailed Pearson's correlation test was performed by SPSS 17.0. As the colors scale shows, the Pearson correlation coefficient (r) presents inside the grid, the yellow color indicates a positive correlation, and the blue color displays a negative correlation. r value (Pearson correlation coefficient) more than 0.4 or less than -0.4 were considered as a strong correlation with positive and negative, respectively.
